# Assessment of Long-Term Sequelae After Severe Malaria: A Retrospective Study

**DOI:** 10.3390/pathogens15020154

**Published:** 2026-01-31

**Authors:** Florian Cardona, Laura Héritier, Sébastien Cortaredona, Coralie L’Ollivier

**Affiliations:** 1 Assistance Publique-Hôpitaux de Marseille, 13005 Marseille, France; 2 IHU Méditerranée Infection, 13005 Marseille, France; 3 Aix-Marseille University, SSA, RITMES, 13005 Marseille, France; 4 Aix-Marseille University, IRD, SSA, Mines, 13005 Marseille, France

**Keywords:** severe malaria, *Plasmodium falciparum*, imported malaria, neurological sequelae, renal impairment, follow-up, patient-reported outcome

## Abstract

Background: Data on long-term sequelae after severe imported *Plasmodium falciparum* malaria in adults are scarce in non-endemic settings. We aimed to quantify early and medium term renal and neurological outcomes and identify prognostic factors. Therapeutic strategies have evolved with widespread intravenous artesunate, yet survivorship data remain limited. Methods: We performed a retrospective study of cases of severe malaria at the University Hospital of Marseille (France) between January 2018 and December 2024. This study is a single-centre retrospective cohort with prospective follow-up using standardised questionnaires. Adults meeting the criteria for severe falciparum malaria were included. The primary endpoint was a composite of renal impairment and/or neurological sequelae assessed at day 28 (D28) and at remote post-discharge follow-up. Patient-reported outcomes were collected at one year. Associations with baseline features were tested using the Fisher’s exact and Wilcoxon–Mann–Whitney tests. Results: Among 474 malaria cases, 66 (13.9%) were severe; of these, 57 met inclusion criteria. Fifty-seven of them were included. All received intravenous artesunate with oral step-down; 35% required ICU care. At D28, 6/41 patients (14.6%) had sequelae (four renal, one neurological, one both). Sequelae at D28 were associated with neurological failure (66.7% vs. 14.3%; *p* = 0.015), severe metabolic acidosis (50.0% vs. 2.9%; *p* = 0.007) and renal impairment at admission (83.3% vs. 2.9%; *p* < 0.001). At remote follow-up, 6/33 patients (18.2%) had sequelae (two renal, three neurological, one both), associated with older age (61.0 ± 5.3 vs. 39.8 ± 15.8 years; *p* = 0.008), D3 blood smear positivity (66.7% vs. 11.5%; *p* = 0.012), neurological failure (66.7% vs. 18.5%; *p* = 0.034) and renal impairment (50.0% vs. 7.4%; *p* = 0.031). No deaths or relapses occurred. At one year, patient-reported outcomes (n = 14) showed persistent symptoms in 8/14, chiefly fatigue and cognitive complaints. Conclusions: In a high-resource, non-endemic setting, renal and neurological sequelae after severe imported malaria are frequent at D28 and persist in nearly one-fifth of cases during post-discharge follow-up. Neurological failure, metabolic acidosis, renal impairment at presentation, older age and D3 blood smear positivity identify patients at risk and support risk-stratified post-discharge follow-up.

## 1. Introduction

According to the World Health Organization (WHO), malaria remains endemic in 85 countries. Two hundred and forty-nine million cases and 608,000 deaths were recorded in 2022, 94% of which occurred in Africa [1]. Mortality affects primarily children under five years of age (67% of deaths). In non-endemic countries, imported malaria remains a challenge in terms of prevention, diagnosis and management. France reports the highest number of imported cases (with an estimated 4995 cases in 2021), about 15% of which are severe, with a case-fatality rate of approximately 2.5% [2]. In these non-endemic settings, post-discharge follow-up is often not standardised, and long-term renal and neurological sequelae in adult survivors may therefore be under-recognised.

Beyond the acute phase, long-term sequelae are documented, particularly among children in endemic settings. One study showed that 7.8% of children with acute kidney injury (AKI) during the index episode progressed to chronic kidney disease (CKD) at one year [3]. Cerebral malaria is associated with deficits in attention [4,5], comprehension and memory [5], as well as motor impairments [6]. A recent meta-analysis also reported a significant increase in language disorders after cerebral malaria [7]. In Gabon, Reiterer et al. described persistent cochlear dysfunction four years after severe malaria in children [8].

Conversely, data in adults are far more limited. Other than the few series focused on cerebral malaria, few studies have examined long-term sequelae. When present, they report alterations in memory and language [9,10,11]. Post-malaria neurological syndrome (PMNS) has long been described [12], classically characterised by a delayed cerebellar ataxia occurring after the acute episode; however, the full clinical spectrum of this syndrome remains poorly defined, particularly in comparison with other post-infectious syndromes (such as long COVID-19) [13,14]. In non-endemic countries, the natural history of long-term sequelae is even less well known as patients are discharged from hospital after receiving antimalarial treatment. Some adult observations suggest persistent MRI lesions and neuropsychological deficits several months after severe malaria [15].

Renal involvement in adults after severe malaria is also heterogeneous. A recent Tanzanian study reported persistent renal impairment at three months in 6.1% of patients who had AKI at diagnosis [16], whereas an ICU series reported complete recovery of renal function among all survivors at follow-up [17]. These discordant findings underscore the need to better characterise renal prognosis beyond hospital discharge. Knowing the long-term effects could enable better post-hospitalisation support and preventive measures to be offered.

In the absence of robust and recent data from non-endemic countries, our objective was to describe, among adults hospitalised for severe *Plasmodium falciparum* malaria in a high-resource setting, the frequency and nature of neurological and renal sequelae at one month after hospitalisation and at longer-term follow-up.

## 2. Materials and Methods

### 2.1. Study Design and Population

We conducted a single-centre retrospective cohort study at the University Hospital of Marseille (France), from 1 January 2018 to 31 December 2024, with prospective collection of long-term sequelae using standardised questionnaires. Eligible participants were all patients who had been treated for severe *P. falciparum* malaria, defined according to the WHO severity criteria in effect at the time of diagnosis. Exclusion criteria were a refusal to participate, vulnerable status at the time of diagnosis (minors, pregnant women or adults under legal protection), a documented history of advanced neurodegenerative disease or the inability to complete the questionnaire in French. The study is reported in accordance with STROBE guidelines, and the completed STROBE checklist is provided in the Supplementary Information (Table S1).

### 2.2. Malaria Diagnosis

Initial diagnosis relied on a Plasmodium-targeted loop-mediated isothermal amplification (LAMP) test (Alethia Malaria, Launch Diagnostic, Paris, France). When positive, a rapid diagnostic test (Palutop4+ Optima, Biosynex, Illkirch-Graffenstaden, France) and thin blood smears were performed to identify the *P. falciparum* species and quantify parasitaemia. Parasitological follow-up was routinely scheduled on day 3 (D3) and day 7 (D7). Assessment at day 28 (D28) was systematically planned but could be missing depending on patient compliance.

### 2.3. Data Collection and Management

Demographic, clinical, laboratory and treatment data were extracted from the electronic medical record (EMR). Baseline variables included age, sex, malaria immunity status, use of chemoprophylaxis, the timescale from symptom onset to emergency department (ED) presentation and from diagnosis to first dose of intravenous (IV) artesunate, antimalarial treatments received (drug, dose, duration) and organ support requirements. Post-discharge healthcare utilisation (outpatient visits, ED presentations, hospitalisations) was retrieved from the EMR and incorporated into the composite outcome during follow-up. All data were entered and managed in a REDCap^®^ database, with controlled access, audit trails and predefined consistency checks. Missing values were subject to verification procedures.

### 2.4. Contact Procedures and Sequelae Assessment via Questionnaire

Eligible patients were contacted using the details recorded in the EMR, by telephone and email. For each participant, at least three contact attempts were made on different days and times. All received an email with the participant information sheet and, when applicable, the questionnaire to return. The questionnaire covered the index hospitalisation, recurrences, post-discharge healthcare utilisation and functional impact (fatigue, dyspnoea, activities of daily living, physical activity). It assessed persistent symptoms at 12 months and neurocognitive and sensory sequelae (attention, memory, motor, sensory, vision, hearing) using dichotomous items and four-level ordinal scales. A final module recorded subsequent exposures and prevention behaviours. It was developed based on the clinical expertise of the different practitioners involved in patient care within our department. The questionnaire is available in the Supplementary Information.

### 2.5. Primary Outcome

The primary endpoint was a composite, assessed at D28 and at long-term follow-up, defined as the occurrence of at least one of the following: renal impairment or neurological sequelae.

### 2.6. Secondary Objective

The secondary objective was to describe, at D28 and at subsequent follow-up, other complications and sequelae related to severe malaria; to identify prognostic factors associated with their occurrence and persistence; and to conduct a qualitative assessment of patient-reported symptoms using a structured questionnaire.

### 2.7. Definitions

Severity was defined according to the French recommendations adapted from WHO [18], including, notably, hyperparasitaemia, non-cardiogenic acute respiratory distress, neurological impairment (Glasgow Coma Scale < 11 or multiple seizures), persistent shock despite fluid resuscitation, coagulopathy or abnormal bleeding, macroscopic haemoglobinuria, AKI, jaundice, hyperlactataemia, metabolic acidosis, hypoglycaemia or severe anaemia.

Malaria immunity status was categorised as “malaria naive,” defined by no prior exposure in an endemic area and no documented history of malaria, and “history of prior malaria episodes,” defined by prior exposure (residence or repeated stays in an endemic area) and at least one documented episode of malaria.

At D28, persistent renal involvement was defined as KDIGO stage ≥ 1 AKI based on serum creatinine (versus reference value) and/or an eGFR < 60 mL/min/1.73 m^2^ not previously known. eGFR was calculated using the CKD-EPI equation. The reference creatinine corresponded, when available, to a measurement prior to the index episode; when unavailable, we used only on eGFR to define renal involvement. At long-term follow-up, renal impairment was considered persistent if these abnormalities remained or progressed to CKD (eGFR < 60 mL/min/1.73 m^2^). The reference creatinine corresponded, when available, to a measurement prior to the index episode.

Neurological sequelae were defined clinically as new or persistent neurological symptoms or signs attributable to the malaria episode, not explained by another cause and present at D28 and/or at long-term follow-up. Patient-reported and/or medically documented “lack of return to baseline” constituted a criterion for sequelae. Transient symptoms lasting less than seven days that resolved before the assessment window were not considered sequelae.

Long-term follow-up was performed between six months and one year after the index episode using the EMR, including clinic visits, ED presentations and hospitalisations within our centre; for each encounter, clinical documentation and available laboratory/imaging data were reviewed to ascertain renal and/or neurological sequelae according to predefined definitions.

### 2.8. Statistical Analysis

Categorical variables are described as counts (percentages) and continuous variables as medians (range or interquartile range). The Fisher’s exact and Wilcoxon–Mann–Whitney tests were used to compare, respectively, proportions and continuous variables between patients with versus without sequelae of severe malaria at D28 and at 6–12 months. Effect sizes for binary variables were expressed as odds ratios (ORs) with exact 95% confidence intervals estimated using Fisher’s exact method. For continuous variables, group differences were quantified using the Hodges–Lehmann estimator with 95% confidence intervals. Given the small sample size and limited number of events, analyses were considered exploratory. Baseline characteristics (age, sex, and disease severity) were compared between patients who remained in the sample and those who were lost to follow-up at Day 28 and at 6–12 months. No statistically significant differences (*p* > 0.05) were observed, suggesting that loss to follow-up was not significantly associated with these key baseline variables.” A table comparing data from patients with follow-up versus lost to follow is provided in the Supplementary Data (Table S2). All analyses were performed with SAS 9.4 (SAS Institute, Cary, NC, USA).

### 2.9. Ethical Considerations

The study received approval from an Ethics Committee (Comité de Protection des Personnes) and is registered on ClinicalTrials.gov (NCT06992297). It was conducted in accordance with the Declaration of Helsinki and applicable French regulations. Written informed consent specific to the follow-up was obtained prior to administering the questionnaires.

## 3. Results

### 3.1. Patient Characteristics

**Figure 1:**
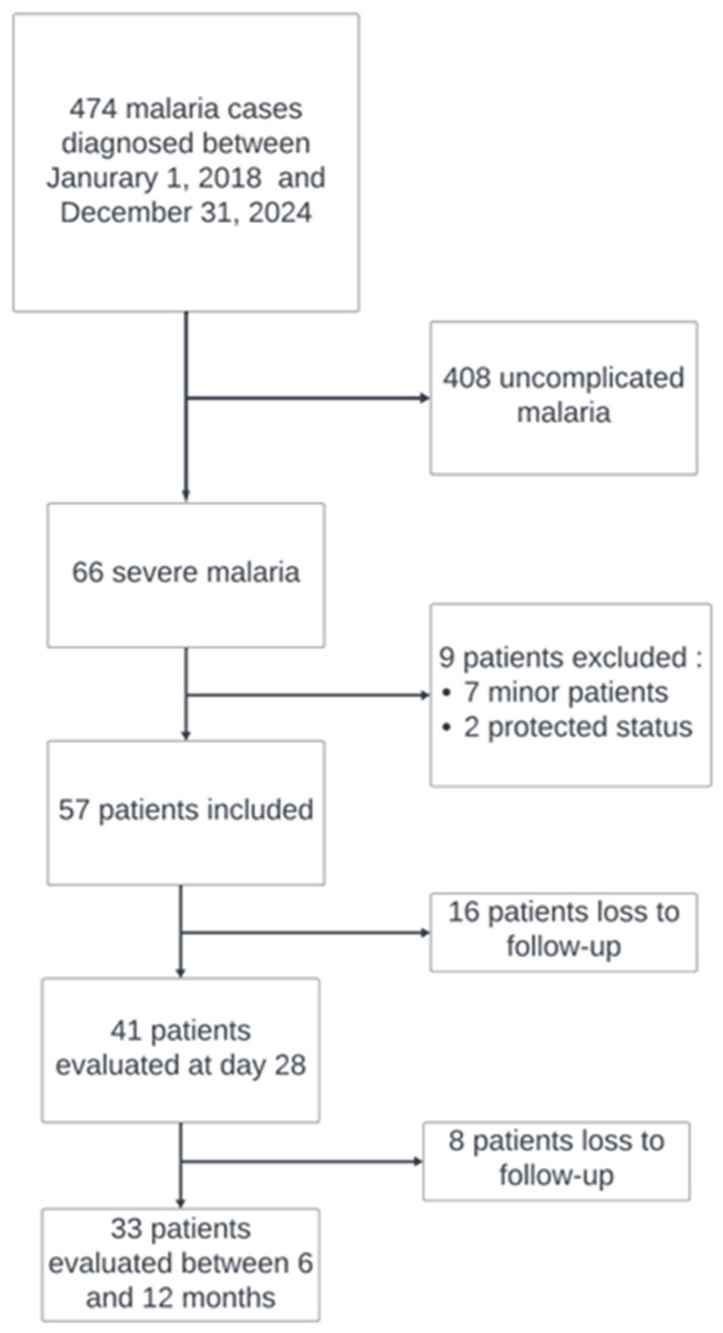
Study flow diagram and follow-up completeness.

Demographic characteristics are presented in Table 1. Over the study period, 474 malaria cases were recorded at our centre. Of the sixty-six patients with severe *P. falciparum* malaria, fifty-seven were included; seven were excluded due to being minors and two because of protected status (one patient under legal guardianship and one pregnant woman). At D28, 41 patients were evaluated and 33 at longer-term follow-up; Figure 1 shows the study flow and follow-up completeness. The median time between symptom onset to ED presentation was four days (IQR 2–6). The vast majority (55/57, 98%) acquired the infection in sub-Saharan Africa. Only one patient had taken complete, guideline-concordant antimalarial chemoprophylaxis. A notable proportion were considered malaria-naive, whereas others had a history of prior malaria episodes.

### 3.2. Severe Malaria Diagnosis

All patients had an initial positive LAMP test with thin blood smears showing the presence of asexual forms. Admission parasitaemia varied widely, with a median of 3.28% parasitised erythrocytes (IQR 0.37–5.15).

### 3.3. Management

All patients received at least three doses of IV artesunate, followed by oral therapy: fifty-one patients (89%) with dihydroartemisinin–piperaquine (Eurartesim^®^) and four (7%) with atovaquone–proguanil (Malarone^®^). One patient received a seven-day course of IV artesunate only, without oral step-down. Overall, 20 patients (35%) were admitted to the intensive care unit (ICU) and 28 (49%) to a step-down (intermediate care) unit; the large majority were subsequently managed on the infectious diseases ward after the intensive phase of care.

### 3.4. Clinical Presentation and Critical Care

In our cohort (n = 57), 29 patients met a single severity criterion, 17 met two, and 11 met three or more. The most frequent severity criteria were parasitaemia higher than 4%, jaundice or bilirubin higher than 50 µmol/L and lactataemia higher than 2 mmol/L. At admission, six patients had renal failure and fourteen had neurologic failure. No episodes of hypoglycaemia were documented. Detailed parameters are presented in Table 2. The median ICU length of stay was four days. Four patients underwent renal replacement therapy, five required endotracheal intubation and three received vasopressors. One patient had *Enterococcus faecalis* bacteraemia, and another developed a pulmonary superinfection

### 3.5. Outcomes and Complications

No deaths or relapses were observed. Several complications were reported: one case of splenic rupture with hemoperitoneum, with favourable outcome without surgery; two cases of retinal ischaemia, one without functional recovery; one case of septic shock complicated by peripheral emboli leading to toe necrosis and subsequent amputation; and two cases of post-malaria neurological syndrome (PMNS), both resolving over several weeks without specific therapy.

### 3.6. Primary Outcome

Of the 57 included patients, 41 (71.9%) had D28 follow-up data; 6/41 (14.6%) had sequelae: four with renal impairment, one with both renal and neurological sequelae, and one with isolated neurological symptoms. In univariable analysis, sequelae at Day 28 were associated with neurological failure (66.7% vs. 14.3%; *p* = 0.015; OR 12.0, 95% CI 1.21–151.8), severe metabolic acidosis (pH < 7.35 or bicarbonate < 15 mmol/L; 50.0% vs. 2.9%; *p* = 0.007; OR 34.0, 95% CI 2.65–436.55) and renal impairment (83.3% vs. 2.9%; *p* < 0.001; OR 170.0, 95% CI 9.11–3172.46). At 6–12 months, sequelae were associated with older age (median 66.0 vs. 38.0 years; *p* = 0.008; Hodges–Lehmann median difference 23 years, 95% CI 8–35), a positive blood smear at Day 3 (66.7% vs. 11.5%; *p* = 0.012; OR 0.07, 95% CI 0.00–0.76), neurological failure (66.7% vs. 18.5%; *p* = 0.034; OR 8.80, 95% CI 0.88–113.08) and renal impairment (50.0% vs. 7.4%; *p* = 0.031; OR 12.50, 95% CI 0.90–186.07) (Table 3).

Of the 57 included patients, 33 (57.9%) had 6–12-month follow-up; 6/33 (18.2%) had sequelae: two with renal impairment, three with neurological symptoms and one with both renal and neurological sequelae. In univariate analysis, sequelae at 6–12 months were significantly associated with older age (61.0 ± 5.3 years vs. 39.8 ± 15.8 years; *p* = 0.008), a positive blood smear at D3 (66.7% vs. 11.5%; *p* = 0.012), neurological failure (66.7% vs. 18.5%; *p* = 0.034) and renal impairment (50.0% vs. 7.4%; *p* = 0.031) (Table 4).

### 3.7. Patient-Reported Long-Term Assessment via Questionnaire

Fourteen patients completed the questionnaire (five by telephone and nine by email). Given the limited response rate, these patient-reported data should be interpreted as descriptive and hypothesis-generating and may not be generalizable to the entire cohort. All recalled a severe malaria episode and an ICU stay. Only one patient experienced a new malaria episode during follow-up.

At one year, 8/14 (57.1%) reported at least one symptom. The median number of symptoms per patient was 1 [0–3] (min–max: 0–8). The most frequent complaints were fatigue/weakness (8/14), followed by myalgia/arthralgia (3/14), shortness of breath/dyspnea (2/14) and night sweats (2/14). In addition, two patients spontaneously reported visual disturbances and two reported memory problems. Six patients consulted a physician, with symptom improvement. Eight patients reported a return to baseline after a median of 105 days (IQR 23–225), whereas three patients indicated they had not returned to their prior baseline.

In the multiple-choice section, nine patients reported fatigue, five of whom experienced it often or very often; three reported dyspnea; three reported disruptions in activities of daily living; five reported memory problems, including three often or very often; and seven reported concentration difficulties. Finally, nine patients returned to endemic areas, and ten reported modifying their travel habits.

## 4. Discussion

In this study, we focused on long-term sequelae in severe imported malaria cases. In our cohort of 57 patients managed in a non-endemic, high-resource setting, we observed a non-negligible frequency of early sequelae (D28: 14.6%), consisting of renal and neurological involvement, with persistence at 6–12 months (18.2%). Notably, mortality was null in our study both at an early stage and during subsequent follow-up. This is likely to be related to the use of IV artemisinin derivatives replacing quinine in our centre for several years. Factors associated with persistent sequelae during follow-up included initial neurological failure (*p* = 0.034), renal impairment at admission (*p* = 0.031), older age (*p* = 0.008) and a positive control blood smear at D3 (*p* = 0.012). Indeed, all patients with renal impairment at D28 or at follow-up had renal impairment at admission. We found no evidence for the effect of country of birth or time to consultation on the occurrence of the primary endpoint. Accordingly, patients with these risk factors should receive intensified clinical and laboratory follow-up to enable early detection of the development or persistence of sequelae. In our centre, 66/474 cases (13.9%) met the criteria for severe malaria, a rate consistent with a 2022 meta-analysis reporting 12.5% severe cases in non-endemic countries [19].

Our findings extend historical data on neurological sequelae after severe imported malaria in adults: Roze et al. previously reported neuropsychological deficits (notably memory) with white-matter MRI lesions and incomplete recovery in half of patients at six months (3/6 full recoveries). In our series, neurological signals persisted in the medium term (6–12 months) for two patients, and two cases of PMNS were identified and counted given the magnitude and duration of clinical symptoms, although both recovered thereafter. Furthermore, the one-year questionnaire captured patient-reported complaints (memory, concentration) consistent with a phenotype of mild cognitive impairment that may persist even in the absence of formal cerebral malaria, particularly in older adults, findings that have been well described in *P. vivax*-endemic areas in the Amazon [20]. These outcomes are biologically plausible through convergent pathways of microvascular dysfunction and endothelial activation, blood–brain barrier disruption, cytokine-driven neuroinflammation (including haemozoin-associated microglial activation), white-matter injury and synaptic/neuronal dysfunction, which may produce subtle yet durable deficits even without neurological symptoms at admission [21]. In patients with hyperparasitemia, hemozoin burden is expected to be high. Hemozoin has been linked to innate immune activation and inflammatory signalling and has also been discussed as a contributor to malaria-associated immune dysfunction [22,23]. These mechanistic considerations provide biological plausibility but were not directly assessed in our cohort.

On the renal side, our observations align with recent Tanzanian data reporting an AKI prevalence of 36% among adults with severe malaria and 6.1% persistent renal impairment at three months, with age, parasitaemia, anaemia and proteinuria as independent predictors. In contrast, a UK intensive care series of patients managed with IV artesunate reported 10% persistent renal impairment at ICU discharge without subsequent persistence, though follow-up criteria were not specified. Renal involvement in malaria is multifactorial and classically results from hypovolaemia, obstruction of the microcirculation by sequestration of parasitised red blood cells and tubular toxicity of haemoglobin-derived pigments released during haemolysis [24]. More recently, it has been shown that activation of the host inflammatory response contributes centrally, at least as much as ischaemic mechanisms, to the pathophysiology of malaria-associated AKI, through endothelial activation [25] and the production of proinflammatory cytokines [26], which promote interstitial oedema and tubular injuries. Glomerulopathies related to malaria, mediated by immune complexes, have also been described [27].

In our cohort, renal function ultimately normalized in patients with later follow-up; however, a subset still met our criteria for renal impairment at D28 and/or during the 6–12-month follow-up window. However, several paediatric studies have shown that AKI during severe malaria is a risk factor for subsequent CKD [3]. Furthermore, serum creatinine is a late and insensitive marker of kidney injury in malaria and tubulo-interstitial lesions may occur without an increase in creatinine, as suggested by biomarker data on “subclinical AKI” [28]. Repeated episodes of local renal inflammation, with or without creatinine elevation, may therefore contribute to the development of CKD in endemic areas. We believe post-malaria renal complications remain under-recognised due to insufficient follow-up beyond the acute phase. An ongoing trial focusing on maladaptive repair pathways in children with AKI during severe malaria, who exhibit apparent recovery after treatment, is expected to better characterise this risk [29].

At one year, more than half of respondents (57.1%) still reported at least one symptom, with very frequent fatigue (9/14, five “often/very often”) and notable cognitive complaints (memory 5/14, concentration 7/14). Despite a median of 105 days to perceived return to baseline, three patients did not feel recovered, suggesting prolonged morbidity after the acute episode. Seventy-one percent (10/14) of respondents reported modifying their travel habits and 9/14 returned to endemic areas, with only one documented reinfection. These findings rely on patient-reported pre- and post-episode assessments without clinical evaluation at the time of reporting, and unfortunately with a low response rate. Consequently, we can not extrapolate the population of patient with severe malaria, but this feedback encourages further investigation of the issue on a broader scale. Persistent fatigue and cognitive complaints after severe malaria may be viewed within a broader post-infectious framework (as described for long COVID), in which persistent inflammation/immune dysregulation and endothelial–microvascular dysfunction are discussed as candidate mechanisms [25,30]. Our data highlight the importance of structured long-term follow-up and a dedicated travel medicine consultation to support safe future travel.

To our knowledge, this is the first study in a high-resource context to explicitly target the short- and medium-term sequelae of severe malaria in adults, combining clinical criteria, laboratory follow-up and a structured questionnaire. Prior work in non-endemic countries has focused mainly on mortality and in-hospital complications [15,17].

The literature suggests that long-term sequelae of severe malaria in adults living in endemic countries may be underestimated and could represent a burden that may increase in the coming years [21,31]. As malaria transmission declines thanks to large-scale prevention and treatment campaigns, the overall incidence decreases, but the age distribution of clinical and severe cases shifts towards older age groups, who have acquired less robust natural immunity than previous generations [32,33]. This epidemiological transition raises the possibility of recurrent severe episodes and a higher risk of persistent neurological and functional sequelae in young adults, a population that has been much less studied than children. Beyond exposure-related immunity, aging is also associated with immunosenescence/inflammaging [34], endothelial–microvascular vulnerability [35] and reduced renal [36] and cerebral functional reserve, which may plausibly increase susceptibility to acute organ injury and limit recovery, thereby increasing the risk of persistent sequelae after severe episodes. At the same time, the progressive disengagement of major donor countries and funding cuts to malaria control programmes increase the risk of resurgence in endemic settings, particularly if vector control and access to effective treatment cannot be sustained [37]. In non-endemic regions, the risk of renewed transmission is compounded by climate change, persistent competent Anopheles vectors and increasing numbers of imported cases, especially in southern Europe, where climatic conditions are becoming more suitable for seasonal transmission and local outbreaks [38,39,40].

Strengths of our study include a homogeneous care pathway with IV artesunate, clinical assessment of sequelae, enhanced follow-up through the EMR and a dedicated questionnaire documenting persistent symptoms at one year. Limitations include the modest sample size, retrospective single-centre design, a non-validated questionnaire, substantial loss to follow-up and a low response rate, exposing the study to selection and recall biases; as an example, the D28 visit was systematically scheduled, incomplete attendance may have caused non-random missing outcome data and long-term sequelae managed outside our centre may have been missed, potentially underestimating sequelae. The sample size was small, particularly at follow-up, with only 41 patients available at D28 and 33 at 6–12 months, and a limited number of events at each time point (n = 6). This constrained statistical power and precluded the use of multivariable regression models, as any such models would have been unstable and at high risk of overfitting. Consequently, analyses were restricted to univariable, exploratory assessments, and results should be interpreted with caution.

Our data support establishing a targeted follow-up pathway for patients at risk after severe malaria: nephrology follow-up and focused neurocognitive evaluation when complaints are present. Multicentre prospective studies with larger samples, renal biomarkers, standardised brain MRI and validated patient-reported outcome measures are needed to refine recovery trajectories and compare the impact of initial severity phenotypes on long-term sequelae.

## 5. Conclusions

In a high-resource setting, adults hospitalised for severe malaria experience non-negligible renal and neurological sequelae, evident at D28 and persisting to 6–12 months, with frequent residual symptoms at one year. Early identification of at-risk patients and organised follow-up are pragmatic levers to reduce the post-malaria functional burden. These findings support the need to consider a standardised follow-up programme after the critical phase.

## Figures and Tables

**Table 1:** Patient characteristics of the study population (N = 57).

	Patient n (%)
**Median** **(IQR)** **age, years**	40 (26–55)
**Sex**	
Male	39 (68)
Female	18 (32)
**Place of Birth ^a^**	
Metropolitan France	20 (36)
Overseas territories	1 (2)
Comoros	2 (4)
Sub-Saharan Africa	32 (57)
Other	1 (2)
**Immunity**	
Malaria naive	23 (40)
History of prior malaria episodes	34 (60)
**Visited malaria-endemic areas**	
Sub-Saharan Africa	55 (100)
Comores	2 (4)
**Anti-malarial chemoprophylaxis**	5 (9)
Optimal adherence	1 (20)
**Median time between symptom onset and ED admission, ^b^ days**	4 (2–6)
**Parasitaemia**	
Less than 4%	25 (44)
Between 4% and 8%	24 (42)
Between 8% and 12%	3 (5)
Greater than 12%	5 (9)
**Intensive care unit (ICU)**	20 (35)
Median (IQR) ICU length of stay, days (IQR)	2.5 (2–5)
**Intermediate care (HDU)**	28 (49)
Median (IQR) HDU length of stay, days (IQR)	1 (1–2)
**General ward hospitalisation**	56 (98)
Median general ward length of stay, days (IQR)	4 (3–6)
**Number of intravenous artesunate injections**	
3 injections	29 (51)
4 Injections	8 (14)
5 injections	13 (22)
6 Injections	3 (5)
9 Injections	4 (7)
**Oral switch**	56 (98)
Primaquine + Dalacine	1 (2)
Malarone	4 (7)
Eurartesim	51 (89)

Abbreviations: ED = Emergency Department; ICU = Intensive Care Unit; HDU = High-Dependency Unit. ^a^: missing data for one patient. ^b^: missing data for one patient.

**Table 2:** Severity criteria at admission.

	Patients n = 57 (%)
Neurological failure: obnubilation, confusion, coma, repeated seizures (≥2/24 h)	14 (25)
Respiratory failure: SaO_2_ < 90% and/or PaO_2_ < 60 mmHg	0
Cardiocirculatory failure	4 (7)
Clinical bleeding	3 (5)
Jaundice or bilirubin > 50 μmol/L	23 (40)
Anaemia < 7 g/dL	2 (4)
Hypoglycaemia < 2.2 mmol/L	0
Metabolic acidosis Ph < 7.35 or bicarbonate < 15 mmol/L	4 (7)
Hyperlactataemia > 2 mmol/L	19 (33)
Parasitaemia > 4%	32 (56)
Renal failure	6 (11)

SaO_2_: arterial oxygen saturation; PaO_2_: arterial partial pressure of oxygen.

**Table 3:** Comparison of patient characteristics according to presence of sequelae at Day 28 (n = 41).

	Presence of Sequelae							
	No (n = 35)		Yes (n = 6)				Total	
	n	%	n	%	*p*-Value ^†^	OR95% CI ^‡^	n	%
**Sex**								
Male	22	62.9	6	100.0	0.152		28	68.3
Female	13	37.1	0	0.0			13	31.7
**Age**								
Median (IQR)	37.0 (24–51)		51.5 (41–66)		0.134		40.0 (26–55)	
≤40	20	57.1	1	16.7	0.093		21	51.2
>40	15	42.9	5	83.3			20	48.8
**Place of birth ^a^**								
Metropolitan France	12	35.3	5	83.3	0.066		17	42.5
Other	22	64.7	1	16.7			23	57.5
**Parasitaemia**								
Less than 4%	16	45.7	3	50	0.109		19	46.3
Between 4% and 8%	16	45.7	1	16.7			17	41.5
Between 8% and 12%	2	5.7	0	0.0			2	4.9
Between 12% and 18%	1	2.9	1	16.7			2	4.9
Greater than 18%	0	0.0	1	16.7			1	2.4
**Day-3 blood smear ^b^**								
Negative	30	88.2	3	50.0	0.055		33	82.5
Positive	4	11.8	3	50.0			7	17.5
**Severity criteria**								
Neurological failure	5	14.3	4	66.7	**0.015**	12.0 1.21–151.8	9	22.0
Cardiocirculatory failure	2	5.7	1	16.7	0.386		3	7.3
Clinical bleeding	3	8.6	0	0.0	1.000		3	7.3
Jaundice or bilirubin > 50 μM	14	40.0	3	50.0	0.679		17	41.5
Anaemia < 7 g/dL	2	5.7	0	0.0	1.000		2	4.9
Metabolic acidosis pH < 7.35 or bicarbonate < 15 mmol/L	1	2.9	3	50.0	**0.007**	34.0 2.65–436.55	4	9.8
Hyperlactataemia > 2 mM	8	22.9	4	66.7	**0.050**	6.75 1.04–43.87	12	29.3
Parasitaemia > 4%	19	54.3	3	50.0	1.000		22	53.7
Renal failure	1	2.9	5	83.3	**<0.001**	170.0 9.11–3172.46	6	14.6
**Time between symptom onset and ED admission ^c^**								
Median (IQR)	4.0 (2–5)		6.5 (4–9)		0.150		4.0 (2–6)	
≤4 days	21	61.8	2	33.3	0.373		23	57.5
>4 days	13	38.2	4	66.7			17	42.5

^a^: missing data for one patient. ^b^: missing data for one patient. ^c^: missing data for one patient; ^†^: Fisher’s exact test or Wilcoxon–Mann–Whitney test. ^‡^: Odds ratio (OR) with exact 95% confidence interval estimated using Fisher’s exact method. ORs are displayed for variables with nominal *p* < 0.05.

**Table 4:** Comparison of patient characteristics according to presence of sequelae during the 6–12 months follow-up (n = 33).

	Presence of Sequelae							
	No (n = 27)		Yes (n = 6)				Total	
	n	%	n	%	*p*-Value ^†^	OR95% CI ^‡^	n	%
**Sex**								
Male	18	66.7	4	66.7	1.000		22	66.7
Female	9	33.3	2	33.3			11	33.3
**Age**								
Median (IQR)	38.0 (24–49)		66.0 (55–66)		**0.** **008**	23.00 8.00–35.00 ^††^	42.0 (28.58)	
≤40	15	55.6	0	0.0	**0.021**	NA	15	45.5
>40	12	44.4	6	100.0			18	54.5
**Place of birth**								
Metropolitan France	9	34.6	4	66.7	0.194		13	40.6
Other	17	65.4	2	33.3			19	59.4
**Parasitaemia**								
Less than 4%	11	40.7	2	33.3	0.906		13	39.4
Between 4% and 8%	11	40.7	3	50			14	42.4
Between 8% and 12%	3	11.1	0	0.0			3	9.1
Between 12% and 18%	2	7.4	1	16.7			3	9.1
Greater than 18%	0	0.0	0	0.0			0	0.0
**Day-3 blood smear ^a^**								
Negative	23	88.5	2	33.3	**0.012**	0.07 0.00–0.76	25	78.1
Positive	3	11.5	4	66.7			7	21.9
**Severity criteria**								
Neurological failure	5	18.5	4	66.7	**0.034**	8.80 0.88–113.08	9	27.3
Cardiocirculatory failure	1	3.7	2	33.3	0.078		3	9.1
Clinical bleeding	1	3.7	0	0.0	1.000		1	3.0
Jaundice or bilirubin > 50 μM	12	44.4	1	16.7	0.364		13	39.4
Anaemia < 7 g/dL	1	3.7	0	0.0	1.000		1	3.0
Metabolic acidosis pH < 7.35 or bicarbonate < 15 mmol/L	2	7.4	1	16.7	0.464		3	9.1
Hyperlactataemia > 2 mM	7	25.9	4	66.7	0.146		11	33.3
Parasitaemia > 4%	16	59.3	4	66.7	1.000		20	60.6
Renal failure	2	7.4	3	50.0	**0.031**	12.50 0.90–186.07	5	15.2
**Time between symptom onset and ED admission ^b^**								
Median (IQR)	3.0 (2–6)		7.5 (0–9)		0.418		4.0 (2–6)	
≤4 days	17	63.0	2	33.3	0.363		19	57.6
>4 days	10	37.0	4	66.7			14	42.4

^a^: missing data for one patient. ^b^: missing data for one patient. ^†^: Fisher’s exact test or Wilcoxon–Mann–Whitney test. ^‡^: Odds ratio (OR) with exact 95% confidence interval estimated using Fisher’s exact method. ORs are displayed for variables with nominal *p* < 0.05. ORs are displayed for variables with nominal *p* < 0.05. ^††^: Hodges–Lehmann estimator with 95% confidence interval. NA: Not applicable.

## Data Availability

The data are not publicly available due to individual privacy. The data presented in this study are available on request from the corresponding author.

## Supplementary Materials

The following supporting information can be downloaded at: https://www.mdpi.com/article/10.3390/pathogens15020154/s1. Supplementary Table S1. STROBE Statement adapted to our study; Supplementary Table S2. Characteristics of patients who were lost to follow-up at Day 28 (n = 41); Supplementary Table S3. Individual description of renal and/or neurological sequelae; Questionnaire using to assess the sequelae in our study.
