# Supplementary material for: Assessment of Long-Term Sequelae After Severe Malaria: A Retrospective Study
Source: Pathogens. 2026 Jan 31;15(2):154. doi: 10.3390/pathogens15020154 (PMC12942715; doi:10.3390/pathogens15020154)
Supplement: Supplementary file 1 [file pathogens-15-00154-s001.zip › pathogens-3955364-supplementary.pdf]

Supplementary Table S1 - STROBE Statement adapted to our study

	Item No	Recommendation	Page No
**Title and abstract**	1	(*a*) Indicate the study's design with a commonly used term in the title or the abstract	1
		(*b*) Provide in the abstract an informative and balanced summary of what was done and what was found	1
**Introduction**			
Background/rationale	2	Explain the scientific background and rationale for the investigation being reported	1
Objectives	3	State specific objectives, including any prespecified hypotheses	1
**Methods**			
Study design	4	Present key elements of study design early in the paper	3
Setting	5	Describe the setting, locations, and relevant dates, including periods of recruitment, exposure, follow-up, and data collection	3/4/5
Participants	6	(*a*) Give the eligibility criteria, and the sources and methods of selection of participants. Describe methods of follow-up	3
		(*b*) For matched studies, give matching criteria and number of exposed and unexposed	
Variables	7	Clearly define all outcomes, exposures, predictors, potential confounders, and effect modifiers. Give diagnostic criteria, if applicable	3/4
Data sources/ measurement	8*	For each variable of interest, give sources of data and details of methods of assessment (measurement). Describe comparability of assessment methods if there is more than one group	3
Bias	9	Describe any efforts to address potential sources of bias	
Study size	10	Explain how the study size was arrived at	3
Quantitative variables	11	Explain how quantitative variables were handled in the analyses. If applicable, describe which groupings were chosen and why	3/4
Statistical methods	12	(*a*) Describe all statistical methods, including those used to control for confounding	4
		(*b*) Describe any methods used to examine subgroups and interactions	
		(*c*) Explain how missing data were addressed	
		(*d*) If applicable, explain how loss to follow-up was addressed	
		(*e*) Describe any sensitivity analyses	
**Results**			
Participants	13*	(a) Report numbers of individuals at each stage of study—eg numbers potentially eligible, examined for eligibility, confirmed eligible, included in the study, completing follow-up, and analysed	7
		(b) Give reasons for non-participation at each stage	
		(c) Consider use of a flow diagram	
Descriptive data	14*	(a) Give characteristics of study participants (eg demographic, clinical, social) and information on exposures and potential confounders	7
		(b) Indicate number of participants with missing data for each variable of interest	7

		(c) Summarise follow-up time (eg, average and total amount)	
Outcome data	15*	Report numbers of outcome events or summary measures over time	10
Main results	16	(*a*) Give unadjusted estimates and, if applicable, confounder-adjusted estimates and their precision (eg, 95% confidence interval). Make clear which confounders were adjusted for and why they were included<br>(*b*) Report category boundaries when continuous variables were categorized<br>(*c*) If relevant, consider translating estimates of relative risk into absolute risk for a meaningful time period	10
Other analyses	17	Report other analyses done—eg analyses of subgroups and interactions, and sensitivity analyses	10
**Discussion**			
Key results	18	Summarise key results with reference to study objectives	14
Limitations	19	Discuss limitations of the study, taking into account sources of potential bias or imprecision. Discuss both direction and magnitude of any potential bias	15/16
Interpretation	20	Give a cautious overall interpretation of results considering objectives, limitations, multiplicity of analyses, results from similar studies, and other relevant evidence	15
Generalisability	21	Discuss the generalisability (external validity) of the study results	15
**Other information**			
Funding	22	Give the source of funding and the role of the funders for the present study and, if applicable, for the original study on which the present article is based	13

Supplementary Table S2 – Characteristics of patients who were lost to follow-up at Day 28 (n=41)

	Lost to follow-up at D28	
	**No (n=41) n (%)**	**Yes (n=16) n (%)**
**Sex**		
Male	28 (68)	11 (69)
Female	13 (32)	5 (31)
**Age**		
≤ 40	21 (51)	8 (50)
> 40	20 (49)	8 (50)
**Place of birth**[a]		
Metropolitan France	17 (42)	3(19)
Other	23 (58)	13(81)
**Median (IQR) time between symptom onset and ED admission**[b]	4 (IQR 2,5–6)	4 (IQR 1–6)
**Parasitaemia**		
Less than 4 %	19 (46)	6 (38)
Between 4% and 8%	17 (42)	7 (44)
Between 8% and 12%	2 (5)	1 (6)
Between 12% and 18%	2 (5)	2 (12)
Greater than 18%	1 (2)	0 (0)
**Day-3 blood smear**[b]		
Positive	7 (17)	4 (25)
Negative	33 (83)	12 (75)
**Intensive care unit (ICU)**		
Yes	13 (32)	7 (44)
**Intermediate care (HDU)**		
Yes	19 (46)	9 (56)
**Severity criteria**		
Neurological failure	9 (22)	5 (31)
Cardiocirculatory failure	3 (7)	1 (6)
Clinical bleeding	3 (7)	0 (0)
Jaundice or bilirubin > 50µM	17 (42)	6 (38)
Anaemia < 7g/dl	2 (5)	0 (0)
Metabolic acidosis pH< 7.35 or bicarbonate < 15mmol/L	4 (10)	0 (0)
Hyperlactataemia > 2mM	12 (29)	7 (44)
Parasitaemia > 4%	22 (54)	10 (63)
Renal failure	6 (15)	0 (0)

a: missing data for one patient. b: missing data for one patient.

Supplementary Table S3. Individual description of renal and/or neurological sequelae

Patient #	Index hospitalization (start–end)	Renal sequelae	Renal severity/definition	RRT (dialysis)	Renal outcome at last follow-up	Time to renal recovery	Neurological sequelae	Neurological description	Symptom onset	Neuro outcome at last follow-up	Time to neuro recovery	Notes
1	22-Oct-2022 – 03-Nov-2022	Yes	AKI KDIGO 3	Yes; 1 session	Recovered (Cr 83 µmol/L; eGFR 90 on 03-Jun-2024)	juin-24	No	NA	NA	NA	NA	Follow-up creatinine values improved over time
2	10-Apr-2020 – 29-May-2020	Yes	AKI KDIGO 3	Yes; 12–27 Apr 2020	Recovered (eGFR 69 in Jan-2021)	janv-21	No	NA	NA	NA	NA	—
3	15-Apr-2019 – 06-May-2019	Yes	AKI KDIGO 3	Yes; 3 sessions	Recovered (Cr 97 µmol/L; eGFR 101 on 13-Sep-2019)	sept-19	No	NA	NA	NA	NA	—
4	19-Apr-2020 – 30-Apr-2020	Yes	AKI KDIGO 3	Yes; CRRT until 24-Apr-2020	Recovered (Cr 92 µmol/L; eGFR 84 at ~3 months)	3 months	No	NA	NA	NA	NA	—
5	18-Apr-2023 – 03-May-2023	Yes	AKI KDIGO 2; eGFR 52 (Aug-2023)	No	Improved / recovered (eGFR 65 in Jan-2024)	janv-24	Yes	Delayed post-malaria cerebellar ataxia (PMNS phenotype)	13-May-2023	Resolved	janv-24	—
6	08-Sep-2023 – 04-Oct-2023	No	NA	NA	NA	NA	Yes	Stuttering + left arm paresthesia; later persistent cognitive/visuospatial difficulties (CT normal; LP negative; MRI planned)	NA	Persistent	Not recovered	Neuropsychological assessment Feb-2025: ongoing deficits
7	15-Jun-2018 – 25-Jun-2018	No	NA	NA	NA	NA	Yes	Confusional speech/word-finding; tremor; cerebellar ataxia	13-juil-18	Resolved	Feb-2019	—
8	10-Feb-2022 – 09-Mar-2022	No	NA	NA	NA	NA	Yes	Visual impairment; bilateral retinal ischemia	08-Feb-2022	Persistent	Not recovered	Visual acuity 1/10 at last follow-up

Abbreviations: AKI, acute kidney injury; KDIGO, Kidney Disease: Improving Global Outcomes; eGFR, estimated glomerular filtration rate (CKD-EPI); RRT, renal replacement therapy; CRRT, continuous renal replacement therapy; PMNS, post-malaria neurological syndrome; Cr, serum creatinine; CT, computed tomography; LP, lumbar puncture; MRI, magnetic resonance imaging; NA, not applicable/ not available. eGFR equation: CKD-EPI.

Questionnaire using to assess the sequelae in our study

Last name – First name

Do you remember having a malaria infection or a malarial episode during your hospitalization?
☐ Yes
☐ No

Do you recall being admitted to an intensive care or critical care unit for this malarial episode?
☐ Yes
☐ No

Have you had another malaria infection since your hospitalization?
☐ Yes
☐ No
Do you remember the year? ………………..

In your opinion, do you feel a deterioration in your general health since your hospital discharge related to this malaria infection?
☐ Yes
☐ No

Which of the following symptoms were still present one year after your discharge from hospital for malaria?
☐ Fever
☐ Cough
☐ Shortness of breath or difficulty breathing
☐ Night sweats
☐ Tiredness or weakness
☐ Loss of smell or taste
☐ Headaches occurring regularly
☐ Muscle or joint pain
☐ Abdominal pain
☐ Frequent diarrhea
☐ Chest pain or tightness
☐ Nausea
☐ Vomiting
☐ Conjunctivitis (i.e., redness of the eyes)
☐ Skin rash
☐ Dizziness or lightheadedness
☐ Loss of appetite
☐ Confusion
☐ Other
☐ I no longer had any symptoms one year after my hospital discharge
Specify other symptom: …………………………

Have you consulted one or more doctor(s) about these sequelae/persistent symptoms after your hospital discharge?
☐ Yes
☐ No

Specify which of the following:
☐ General practitioner
☐ Ophthalmologist
☐ Rheumatologist
☐ Neurologist
☐ Gastroenterologist
☐ Cardiologist
☐ Psychiatrist
☐ Psychologist
☐ Physiotherapist
☐ Other
Specify other doctor: ……………………………………

Did this/these appointments help reduce or improve the sequelae/symptoms?
☐ Yes
☐ No

After how long did you return to your physical condition prior to your hospitalization?
……………………………………………………………………

Do you feel fatigue since your hospitalization for malaria?
☐ Yes, very often (for example, you feel tired most of the time, even after a good night's sleep)
☐ Yes, often (for example, you feel tired several times a day)
☐ Yes, sometimes (for example, you feel tired a few times a week)
☐ No (you do not feel excessive fatigue)

Do you feel short of breath since your hospitalization for malaria?
☐ Yes, very often (for example, you become short of breath after light activities such as walking a short distance)
☐ Yes, often (for example, you become short of breath several times a day)
☐ Yes, sometimes (for example, you become short of breath a few times a week)
☐ No (you do not notice any unusual shortness of breath)

Since your hospitalization for malaria, do you have difficulties performing your daily activities?
☐ Yes, very often (for example, you need help with simple tasks such as getting dressed or preparing meals, going shopping)
☐ Yes, often (for example, you have difficulties with certain tasks but can manage most daily activities)
☐ No (you do not have difficulties performing your daily activities)

Do you engage in sports activities since your hospitalization for malaria?
☐ Yes, regularly (for example, several times a week)

☐ Yes, from time to time (for example, a few times a month)
☐ No, I no longer play sports since the hospitalization
☐ No, I did not play sports before the hospitalization either

Have you consulted a psychologist since your hospitalization for malaria?
☐ Yes, regularly (for example, you have therapy sessions several times a month)
☐ Yes, sometimes (for example, you have consulted a psychologist a few times since your hospitalization)
☐ No (you have not felt the need to consult a psychologist)

Do you have attention difficulties that have appeared since your hospitalization for malaria?
☐ Yes, very often (for example, you have difficulty concentrating on a task, even a simple one, most of the time)
☐ Yes, often (for example, you have difficulty concentrating several times a day)
☐ Yes, sometimes (for example, you have difficulty concentrating a few times a week)
☐ No (you do not notice problems with concentration)

Do you have memory problems that have appeared since your hospitalization for malaria?
☐ Yes, very often (for example, you frequently forget things, even simple ones)
☐ Yes, often (for example, you forget things several times a day)
☐ Yes, sometimes (for example, you forget things a few times a week)
☐ No (you do not notice memory problems)

Do you have difficulty controlling your movements or the impression of having lost strength since your hospitalization for malaria?
☐ Yes, very often (for example, you have trouble holding objects or performing precise movements regularly)
☐ Yes, often (for example, you have trouble controlling your movements several times a day)
☐ Yes, sometimes (for example, you have trouble controlling your movements a few times a week)
☐ No (you do not notice any loss of control or strength)

Do you have touch disturbances or tingling sensations in your limbs that have appeared since your hospitalization for malaria?
☐ Yes, very often (for example, you regularly feel tingling or numbness in your limbs)
☐ Yes, often (for example, you feel tingling several times a day)
☐ Yes, sometimes (for example, you feel tingling a few times a week)
☐ No (you do not notice any abnormal sensations in your limbs)

Do you have vision problems that have appeared since your hospitalization for malaria?
☐ Yes, very often (for example, you regularly have blurred or double vision)
☐ Yes, often (for example, you have blurred vision several times a day)
☐ Yes, sometimes (for example, you have blurred vision a few times a week)
☐ No (you do not notice any change in your vision)

Do you use glasses or contact lenses since your hospitalization for malaria?

☐ Yes, since the hospitalization (for example, you did not need glasses before)
☐ Yes, but I was already using them before the hospitalization
☐ No

Do you have hearing problems that have appeared since your hospitalization for malaria?
☐ Yes, very often (for example, you regularly hear non-existent sounds, or you have difficulty hearing conversations)
☐ Yes, often (for example, you have difficulty hearing several times a day)
☐ Yes, sometimes (for example, you have difficulty hearing a few times a week)
☐ No (you do not notice any change in your hearing)

Do you use a hearing aid since your hospitalization for malaria?
☐ Yes, since the hospitalization (for example, you did not need a hearing aid before)
☐ Yes, but I was already using it before the hospitalization)
☐ No

Before your trip, did you have a consultation dedicated to travel/travel medicine?
☐ Yes
☐ No

Have you returned to a malaria-affected area since your malarial episode?
(For example, on the African or Asian continent, or in South America)
☐ Yes
☐ No

Have you taken preventive treatment against malaria?
(For example, Malarone, doxycycline, mefloquine)
☐ Yes
☐ No

Has this episode made you change your habits when you travel?
(For example, by taking additional precautions such as mosquito nets, repellents, etc.)
☐ Yes
☐ No
